# Study of the Effect of Phosvitin as a Potential Carrier on the Permeation Process of Somatotropin (STH) and Corticotropin (ACTH) from Biodegradable Polymers Used as Vehicles for STH and ACTH in Semi-Solid Formulations for Skin Application

**DOI:** 10.3390/polym16182640

**Published:** 2024-09-18

**Authors:** Wioletta Siemiradzka, Karolina Kędzierska, Wojciech Rynk, Barbara Dolińska

**Affiliations:** Department of Pharmaceutical Technology, Faculty of Pharmaceutical Sciences in Sosnowiec, Medical University of Silesia, Kasztanowa 3, 41-200 Sosnowiec, Poland

**Keywords:** biodegradable polymers, functional properties of polymers, phosvitin, skin permeation, drug carrier, hydrogels, topical application, natural membrane, artificial membrane, rheological parameters

## Abstract

Phosvitin shows chelating abilities, an affinity for ACTH (corticotropin), growth factors, antioxidant properties, and acidic nature. An attempt was made to use this protein in hydrogels as a transporter of other protein substances: somatotropin (STH) and (ACTH). The aim of the study was to evaluate the effect of phosvitin on the permeation of ACTH and STH from semi-solid forms of the drug applied to the skin. Four hydrogel substrates were prepared using natural polymers: sodium alginate, methylcellulose, and starch. Based on the evaluation of physicochemical parameters, the hydrogel with the most favorable properties was selected and loaded with the active substances STH and ACTH, followed by the addition of phosvitin. A study of the permeation of STH and ACTH through the artificial cellulose membrane and through porcine skin was carried out without and with the addition of phosvitin. The effect of protein substances on rheological and textural parameters was studied. The evaluation of physicochemical parameters showed a favorable effect of STH and Phosvitin on the stability of the hydrogel with 4% methylcellulose and no effect of ACTH. All prepared formulations showed a reaction close to the natural pH of human skin. In the porcine skin permeation study, the addition of Phosvitin to the hydrogel with STH caused a slight increase in the amount of STH permeated and an increase in the time for STH to permeate porcine skin by 30 min. Phosvitin caused an increase in the amount of ACTH permeated through porcine skin almost twofold. Phosvitin may prove to be a promising permeation promoter for model protein-peptide substances when applied to the skin surface.

## 1. Introduction

In recent years, interest in the use of hormones in medicine has increased. Hormones are substances that are sensitive to the gastrointestinal environment, and oral preparations do not provide an adequate therapeutic effect. So far, somatotropin (STH) preparations, which were introduced for subcutaneous treatment in the 1980s, are known [1]. Somatostatin analogs are manufactured in injectable form for intramuscular and subcutaneous administration [2]. Corticotropin (ACTH) is used in the form of an injectable subcutaneous or intramuscular extended-release formulation, Acthar gel, in which ACTH is dissolved in a gelatin solution [3].

The utilization of hormones in the form of topical preparations represents a promising avenue for the supplementation of hormone deficiencies, circumventing the discomfort commonly associated with the administration of injections. There are studies confirming the skin penetration of protein-peptide particles in the range of 4.5–65 kDa. It has been found that the transport of these substances does not depend solely on the size of their particles. The penetration of substances is influenced by many factors such as the nature of the molecule, its physicochemical properties, the type of matrix in which the active substance is incorporated, as well as the condition, state, and thickness of the skin and the age of the patient [4,5,6].

Another promising protein seems to be phosvitin. Phosvitin (PSV) is the major phosphoprotein found in the egg yolk. It is a source of amino acids necessary for proper body function. Phosvitin has anti-inflammatory and antioxidant properties. It inhibits the production of pro-inflammatory cytokines and has antimicrobial activity against gram-negative bacteria. In addition, it can affect cell division, suggesting anticancer activity. It has a beneficial effect on the immune system by increasing the activity of T-lymphocytes and the number of cytokines produced [7,8].

The use of phosvitin in skin care products protects against free radicals. Phosvitin inhibits the activity of enzymes that degrade the ECM (extracellular matrix) so that the aging process is inhibited. In addition, the use of phosvitin helps maintain healthy skin by increasing collagen production and inhibiting melanogenesis [8].

The large number of phosphoryl groups and the small proportion of hydrophobic amino acids make phosvitin predominantly hydrophilic, with only 10% of the amino acids in its structure being hydrophobic. Phosvitin is an acidic protein rich in phosphate groups, mainly bound by serine. In acidic solutions of low ionic strength, phosvitin has good metal chelating potential. It is able to form complexes with divalent ions such as Ca^2+^, Mg^2+^, Co^2+^, Mn^2+^ and with Fe^3+^ [9]. Therefore, it is used in dietary supplements to increase the bioavailability of iron, especially in patients with iron deficiency or anemia [7]. Phosvitin protects against lipid oxidation by binding heavy metal ions [10]. In addition, phosvitin shows better emulsifying activity and emulsion stability compared to other proteins such as bovine serum albumin, β-casein, or soy protein [7]. Phosvitin peptides added to diets at a concentration of 0.125–0.5% (1.25–5.0 mg/g) significantly increase Ca absorption and accumulation in bone [11].

Somatotropin (STH), also designated as growth hormone (GH), is a polypeptide hormone predominantly produced by the anterior lobe of the pituitary gland. STH is a protein polypeptide hormone comprising 191 amino acids. Two isoforms of 22 kDa and 20 kDa are known to exist within the body [12]. The half-life of GH in the blood is relatively short, at 20–50 min, and this is due to its pulsatile secretion [13].

The primary stimuli that stimulate GHRH (growth hormone-releasing hormone) secretion are low GH concentrations, stress factors, sleep, exercise, nutrition, glucose or glucocorticosteroids, fatty acids, insulin, amino acids, and sex hormones [14]. The stimuli that stimulate the secretion of GHIH (growth hormone inhibitory hormone) are high concentrations of GH, as well as glucose, amino acids, and sex hormones. The most important factor regulating the secretion of insulin-like growth factor (IGF-1) is the pulsatile secretion of GH [15].

The primary function of GH is to stimulate growth by stimulating chondrocyte proliferation, as well as regulating lipolysis and maintaining calcium homeostasis and bone health. With GH, the body is able to increase physical and mental activity. GH is also involved in regulating immune system function [15]. The action of GH is through the stimulation of insulin-like growth factor (IGF-1) production in the liver, which subsequently stimulates growth.

An imbalance in the production of GH within the human body can result in the development of various metabolic disorders and diseases. Excessive GH production can result in gigantism, whereas GH deficiency can lead to insulin resistance and an increased incidence of impaired glucose tolerance and diabetes, as well as dwarfism [16].

Since 1985, recombinant human growth hormone (GH) has been employed in the treatment of various conditions in children and adults associated with growth hormone deficiency [17]. Growth hormone has been employed to facilitate growth in individuals with a range of low-growth conditions that are not directly associated with GH deficiency (GHD) such as Turner syndrome, Noonan syndrome, Prader–Willi syndrome, idiopathic growth hormone deficiency, chronic renal failure, and children born with low body weight relative to gestational age [18,19].

The symptoms of GH deficiency in adults may include a decline in mood and general well-being, loss of muscle mass, increased body fat, hyperlipidemia, bone weakness, or an increased predisposition to atherogenesis [20]. Adults with GHD are more susceptible to developing non-alcoholic steatohepatitis (NASH) and non-alcoholic fatty liver disease (NAFLD) [21]. Substitution therapy has been demonstrated to positively influence muscle and fat mass, the lipid profile, lower diastolic blood pressure, and enhance bone mass. Furthermore, the therapy has been shown to improve patients’ quality of life [20].

STH has been successfully employed in the treatment of a number of diseases that result in muscle mass loss, including HIV (human immunodeficiency virus). It has also been utilized in the management of catabolic diseases such as cystic fibrosis and inflammatory bowel disease (IBD), which encompasses Crohn’s disease and ulcerative colitis. One significant application of STH is its impact on bone metabolism and the treatment of arthritis. Growth hormone has been demonstrated to facilitate repair processes in bone tissues, thereby stimulating bone growth and mineralization. Furthermore, STH also affects the aging process, plays a role in neurological rehabilitation, and alleviates inflammatory bowel disease. Additionally, it can promote wound and burn healing, relieve the symptoms of fibromyalgia, improve hypertension control, and prevent osteoporosis in the postmenopausal period. All of the aforementioned functions of STH include the ability to regenerate tissue, independently of GHD [18].

Adrenocorticotropic hormone (ACTH) is a peptide hormone with a molecular weight of 4.5 kDa. It is produced by the anterior lobe of the pituitary gland and also by the placenta, brain, skin, and adrenal medulla. It is secreted by the anterior lobe of the pituitary gland [22,23,24]. ACTH secretion is primarily regulated by two hypothalamic hormones: corticotropin-releasing hormone (CRH) and arginine vasopressin (AVP) [24]. Its primary function is the regulation of cortisol production [23]. However, it also plays a role in other physiological processes. Due to its proteolytic degradation, its half-life in the body is relatively short [24].

In their research, Robinson and Pickering explored the potential of the newest class of synthetic peptides, melanocortins, in the prevention and treatment of diabetes complications. Melanocortins have been demonstrated to be well tolerated in early animal and human studies for the treatment of a range of associated with the etiology and complications of diabetes. They may exert beneficial effects in these contexts. Further studies are needed to ensure the specificity of melanocortin receptor activation and to modify the formulation of the drug to maximize the serum half-life, with the aim of advancing the development of melanocortins for the treatment of diabetes and its complications [25].

Despite their low permeability across the brain barrier, both ACTH and MSH (Melanocyte-Stimulating Hormone) have been observed to stimulate nerve cell regeneration, exert antiepileptic effects, and influence social behavior. In the kidney, ACTH has been demonstrated to reduce tumor necrosis factor-induced tubular necrosis, exert immunomodulatory effects, and protect podocytes from apoptosis. ACTH has been demonstrated to enhance collagen synthesis in osteoblasts and to regulate thymus growth. It is notable that the effects of all melanocortins on immune cells are not insignificant. They exert immunomodulatory and anti-inflammatory effects through a variety of mechanisms, as evidenced by studies [23,26]. Melanocortin receptors are also present in osteoblasts, osteoclasts, chondrocytes, and fibroblasts, thereby enabling the use of ACTH in the treatment of rheumatoid arthritis and other diseases with a similar course and localization. The most well-documented application of ACTH is in the treatment of multiple sclerosis [27,28,29,30]. It has been demonstrated that ACTH exerts a beneficial effect in the treatment of nephrotic syndrome, a condition characterized by hypoalbuminemia, edema, proteinuria, and hyperlipidemia [31,32]. A review of the literature over the past 10 years reveals that the kidney is an important target organ for ACTH, both in terms of clinical efficacy and molecular biology mechanism. A greater number of studies have been conducted on the use of ACTH in the treatment of nephrotic syndrome in adults than in children, irrespective of the underlying pathology and the methodology employed. The results consistently demonstrate a reduction in proteinuria and the preservation of renal function. ACTH has been demonstrated to be an effective agent for the control or elimination of ocular inflammation, even in cases where the condition has proven resistant to conventional steroids or other therapeutic interventions. The occurrence of side effects associated with ACTH is uncommon, with the highest probability of such effects manifesting at relatively elevated doses of ACTH [33]. The efficacy of ACTH has been demonstrated in the treatment of gout and pyrophosphate arthritis [28]. For the treatment of acute gout, interleukin-1 blockers may offer a promising avenue for patients with multiple comorbidities. However, these agents are expensive and can cause immunosuppression. ACTH is widely available in many European countries and may be an alternative for the treatment of acute gout in patients with coexisting infections or in cases where septic arthritis has not been completely ruled out. ACTH has a proven good safety profile and can be used in selected patients with multiple comorbidities for the treatment of gout [34].

The objective of this study was to ascertain the impact of incorporating the protein phosvitin on the permeation of STH and ACTH across both artificial (cellulose membrane) and natural (porcine skin) membranes. Additionally, the physicochemical properties of the prepared hydrogels were examined.

The hydrogel substrates were prepared using a selection of natural polymers, including sodium alginate, methylcellulose (MC), starch, and glycerol. The rheological, textural, pH, spreadability, and sensory evaluations were conducted and analyzed. Following preliminary analysis of these properties, the hydrogel exhibiting the most favorable physicochemical parameters was selected for the loading of active substances. The substances under investigation were STH and ACTH. Given the properties of phosvitin, including its chelating ability, affinity for ACTH, growth factors, and antioxidant properties, as well as its acidic character, an attempt was made to utilize it in the prepared hydrogels with STH and ACTH as a transporter of active substances. To this end, a permeation study of STH and ACTH was conducted, and the impact of the addition of phosvitin on the permeation process through an artificial membrane (cellulose membrane) and a natural membrane (porcine skin) was evaluated.

## 2. Materials and Methods

### 2.1. Hydrogels Preparation

A total of four hydrogel vehicles, comprising different gelling agents were prepared. To prepare a hydrogel with sodium alginate (ALG) (Biomus, Lublin, Poland), the first step was to prepare a 0.5% solution of calcium chloride CaCl_2_ (Chempur, Piekary Śląskie, Poland) and add it to the previously mixed glycerol and water. Subsequently, sodium alginate was then added in small quantities and vigorously stirred at room temperature until a viscous hydrogel of the desired consistency was formed. The glycerol hydrogel (GLY) was prepared by creating a suspension of wheat starch (PPH, Galfarm, Kraków, Poland) in water for injection (Fresenius KABI, Błonie, Poland). This suspension was then introduced into glycerol (Microfarm, Zabierzów, Poland) that had been heated to 60 °C prior to use. The mixture was heated until it reached a viscosity that was sufficiently thick.

MC-based substrate (viscosity of 2% solution at 25 °C: 3755 mPa·s; Sigma Aldrich, Inc., St. Louis, MO, USA) was prepared at a concentration of 4% (4% MC) and 6% (6% MC). Water together with glycerol was heated to 80 °C. Then, MC was added in small portions, stirring vigorously with a glass baguette until the proper consistency was reached. The bases were subsequently stored in a refrigerator set at 4 °C. In order to obtain transparent hydrogels with MC, it was necessary to place them in the refrigerator for a minimum of 24 h prior to commencing further work with these substrates.

The hormones, STH (Genotropin 12-Somatotropin, Powder for preparation of solution for injection 12 mg (36 IU), batch number GF1722, Pfizer, Europe MA EEIG, Bruxelles, Belgium), and ACTH (freeze-dried, Biochefa, Poland) were introduced into the hydrogels in the form of solutions—aqueous (STH) and glycerol (ACTH)—after the substrates were prepared and brought to room temperature and stirred until they became thickened. Phosvitin (Sigma-Aldrich, USA) was introduced at the end into hydrogels loaded with STH or ACTH, respectively, in an amount corresponding to the final concentration in the formulation of 0.5% relative to ACTH and STH. This was completed in the form of an aqueous solution with a concentration of 1 mg/mL. The composition of the prepared hydrogels, which were utilized as matrices for protein-peptide substances and hydrogels containing STH and ACTH as active substances, is presented in Table 1. All hydrogels were stored in a refrigerator at 4 °C.

### 2.2. Testing the Spreadability of Hydrogel Bases

In order to test the spreadability of the obtained hydrogel bases, a methodology was employed which utilized two plates and a set of weights. A volume of 0.5 cm^3^ of hydrogel was applied to the glass plate using a syringe. Subsequently, the second plate was positioned on top of the initial plate. Following the application of the load to the sample, the radius of the stretched hydrogel was recorded at 30-s intervals using a scale with a coordinate system plotted on it. Subsequently, weights of 50 g, 100 g, 200 g, 300 g, 400 g, and 500 g were added. Following the application of each weight, the radii of the stretched hydrogels were recorded on the axes of the coordinate system. The mean value of the recorded radii was then employed to calculate the surface area of the stretched ointment. Hydrogel samples for determination were applied to a glass plate on three occasions at room temperature.

The results obtained permitted the calculation of the area occupied by the prepared hydrogels in accordance with the formula:

(1) $$P=\pi r^{2}$$

where *P*—surface area occupied by the hydrogel (m^2^), *r*—radius of the hydrogel (cm).

### 2.3. Sensory Analysis

The sensory evaluation of the prepared hydrogel bases, comprising 4% ALG, GLY, 4% MC, and 6% MC, was conducted in accordance with the established guidelines by A.M. Pens’e-Lh’eritier [35].

In order to evaluate the preparations, a series of characteristics were selected for analysis, including uniformity, consistency, cushion effect, adhesion, and the ability of the preparation to spread on the skin and stickiness. The selected sensory parameters, along with the determination of their severity, are presented in Table 2 for reference. To assess uniformity and smoothness, approximately 0.5 cm^3^ of the preparation was applied to the slides and then distributed evenly in a circular motion. The presence of lumps or air bubbles was noted. The consistency was evaluated by dipping a finger at an angle of 45° to 60° into the preparation container and then rapidly withdrawing it. The resistance of the cream when the finger was immersed and the contact between the finger and the hydrogel when the finger was pulled out was also observed and recorded. The term “cushion effect” was used to describe the amount of product that could be felt between the fingers when rubbing them together. To evaluate the product’s adhesion properties, it was retrieved from its container with the finger. The product demonstrated good adhesion when it could be picked up with ease, without spillage, and formed a permanent, characteristic cone on the fingertip. The resistance of the hydrogel during spreading was evaluated to determine the ease of application and the even distribution of the product on the skin. To evaluate the product’s stickiness, it was applied with a finger to a selected area of the skin. After a brief interval, the hand was pressed against the lubricated area to ascertain whether the product retained its adhesive quality on the skin. To assess whether the test product leaves an oily film on the skin, a test was conducted by applying the product to the skin and observing if an oily film was formed.

### 2.4. A Study of the Rheological Properties of Hydrogels

The rheological properties of the prepared hydrogels were determined using a Lamy RM 200 TOUCH rotational rheometer (Lamy Rheology Instruments, Champagne au Mont d’Or, France), controlled by Rheomatic software (software from Lamy Rheology Instruments, Champagne au Mont d’Or, France). For the measurements, a CP 2445 plate-to-plate measuring spindle system and a CP-1 Plus thermostat were utilized. Prior to the measurements, the samples were removed from the refrigerator and placed on the bottom plate of the thermostatic system after 30 min. The determinations were made at a temperature approximating that of human skin, which is 32.0 ± 0.5 °C.

Viscosity measurements of the hydrogel were conducted at three distinct shear rates. The measurements were conducted at shear rates of 30 s⁻^1^, 60 s⁻^1^, and 100 s⁻^1^. Flow curves were determined via the flow test method in the shear rate range D = 5–200 s^−1^, with a measurement time of 30 s. Additionally, a stepwise measurement of shear stress versus shear rate was conducted using the Step-by-Step method. The test was conducted on hydrogel bases that had been unloaded with active substances (4% ALG, GLY, 4% MC, and 6% MC) and on preparations based on 4% MC that contained STH and STH with the addition of phosvitin, as well as ACTH and ACTH with the addition of phosvitin.

### 2.5. Texture Analysis

A texture analysis of the test preparations was conducted using a TX-700 Lamy Rheology texture-meter (Lamy Rheology Instruments, Champagne au Mont d’Or, France), employing the TPA (Texture Profile Analysis) test. The test was conducted at room temperature. A cylindrical steel sensor was utilized for the measurement, which was dipped twice into each sample at a speed of 1 mm/s to a depth of 10 mm. Based on the resulting texture profiles, the hardness, cohesiveness, adhesiveness, and elasticity of the prepared hydrogels (4% ALG, GLY, 4% MC, 6% MC, and 4% MC/STH, 4% MC/STH/PSV, 4% MC/ACTH, and 4% MC/ACTH/PSV) were determined.

### 2.6. Determination of pH Value

To ascertain the pH of the prepared hydrogel preparations, a potentiometric method was employed in accordance with the standards set forth in the Polish Pharmacopoeia XII [36]. The measurement was conducted by directly immersing a glass electrode (In Lab Expert Pro-ISM, No. 30014096, Mettler-Toledo AG, Greifensee, Switzerland) in the hydrogel.

### 2.7. Study of the Permeation of STH, STH with Phosvitin, ACTH, and ACTH with Phosvitin through a Cellulose Membrane

#### 2.7.1. Model Membranes

Two types of membranes were utilized in the experiment, an artificial cellulose membrane with a pore size of 100 kDa (Spectra/Por^®^ Dialysis Membrane, Biotech CE, MWCO: 100 kDa Spectrum Laboratories Inc., California, CA, USA) and a natural membrane-porcine skin.

The artificial membrane was immersed in injection water for 30 min prior to testing. The model natural membrane, porcine skin, was obtained from a local farmer. Skin sections were trimmed to a diameter of 2.5 ± 0.5 cm^2^. Each sample was spread over the skin surface. The preparation of porcine skin is described in detail by Siemiradzka et al. [37].

#### 2.7.2. Determination of STH and ACTH

The methodology employed for the measurement of STH and the associated permeation study was meticulously delineated in the seminal paper by Siemiradzka, which explored the permeation of STH through the skin [4]. The measurements were conducted using a UV-VIS Cecil CE 3021 spectrophotometer (Cecil Instruments Limited, Cambridge, UK). The amount of STH permeated was quantified using a curve with the equation y = 0.8953x + 0.0032; R^2^ = 0.999, with measurements taken spectrophotometrically at λ = 276 nm, employing the PBS solution as a reference. The mean value was calculated based on six replicates. The method of ACTH determination was previously described by Siemiradzka et al. [38,39]. The ACTH content was subsequently calculated from the standard curve, which yielded the following equation: y = 0.6925x − 0.0123; R^2^ = 0.9996; *p* < 0.01 [38,39].

#### 2.7.3. Permeation Test

The permeation of STH and ACTH from a 4% MC-based hydrogel substrate was studied using Franz flow cells, which were made of white borosilicate glass and provided with a stirrer (type 03, 9 mm, sheathed, flat connector, type 02, 5 mL) and in/out ports in the receptor chamber (LPP Equipment Warsaw, Warsaw, Poland). A three-station stirrer for Franz cells with an anodized aluminum handle (LPP Equipment Warsaw) and a recirculating thermostat for the Franz diffusion cell system (LPP Equipment Warsaw) were utilized.

Following the application of the preparations to the membrane in an amount of approximately 0.5 g, the chamber was hermetically sealed in order to prevent the evaporation of the acceptor fluid. A capillary tube was used to introduce 5 mL of phosphate buffer pH = 7.4 into the system. The buffer was composed of the following components: sodium chloride p.d.a. (Chempur, Piekary Śląskie, Poland), potassium dihydrogen phosphate (Chempur, Piekary Śląskie, Poland), and disodium hydrogen phosphate anhydrous (Chempur, Piekary Śląskie, Poland, water).

Samples of 2 mL were collected at designated intervals (0.5, 1, 1.5, 2, 2.5, 3, 4, 5, 6, 7, and 8 h). Each sample was supplemented with a new portion of phosphate buffer. Subsequently, the absorbance of the collected samples was quantified using a spectrophotometer at λ = 276 nm (for STH) and λ = 276.5 nm (for ACTH). To confirm the absence of any effect of phosvitin on the accuracy of STH and ACTH determinations, absorbance was also measured at λ = 271 nm, in accordance with the determined absorption maximum of phosvitin. The reference solution was PBS with a pH of 7.4. Six replicates (n = 6) were performed for each sample.

The correlation coefficients of the permeation kinetics of the test substance were determined using the following models: the Higuchi model, the Korsmeyer-Peppas, and first-order models [40].

### 2.8. Stability Study

The stability of the hydrogels was evaluated through a biotechnology/biological products’ stability test, conducted under the conditions specified by the International Council for Harmonisation (ICH) and the Q5C guideline.

The temperature was maintained at 25 ± 1 °C and 5 ± 3 °C. The samples were stored for a period of four weeks [41]. The contents of active substances, pH, and viscosity were monitored and documented in the prepared hydrogels. The contents of the active substances, namely STH and ACTH, were determined by spectrophotometry using a CECIL apparatus (UV-VIS Cecil CE 3021 Instruments Limited, Cambridge, UK) at wavelengths λ1 = 276 nm (STH) and λ2 = 276.5 nm (ACTH). A Lamy RM 200 Touch rotational rheometer (Lamy Rheology Instruments, Champagne au Mont d’Or, France) was employed for rheological testing. The pH was determined by directly immersing a glass electrode (In Lab Expert Pro-ISM, No. 30014096, Mettler-Toledo AG, Greifensee, Switzerland) in the hydrogel.

### 2.9. Statistical Analysis

The mean values with standard deviation were calculated and subjected to statistical analysis using the Microsoft Excel package and the StatSoft, Inc. (Kraków, Poland) Statistica option. Industrial analysis and experimental design (DOE) were employed. The Statistica Pharmaceutical Kit: Statistica “Release Profiles” was utilized to analyze the results of STH and ACTH permeation from the hydrogels, as well as the similarity and difference factors of the f1 and f2 methods. The level of statistical significance was calculated at *p* < 0.05 using Student’s *t*-test.

## 3. Results

### 3.1. Spreadability Test

**Figure 1:**
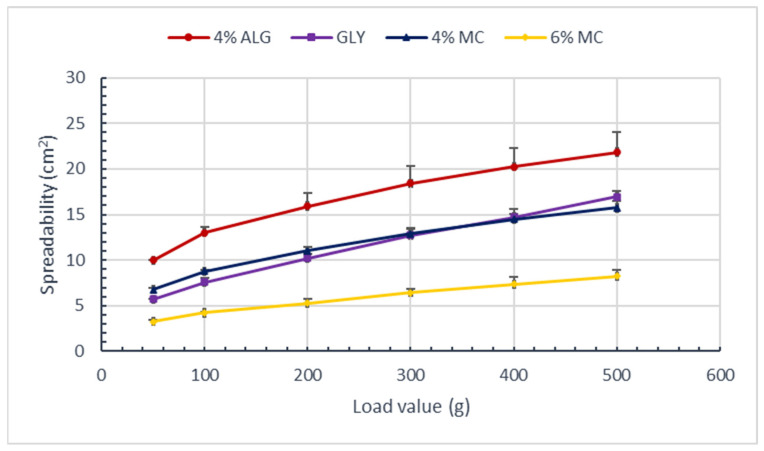
Values of hydrogel surface area depending on the applied load; (n = 3).

The results of the hydrogel spreadability test, conducted with 4% ALG, GLY, 4% MC, and 6% MC are presented in Figure 1.

The 4% ALG-based substrate exhibited the greatest spreadability, while the 6% MC-based hydrogel demonstrated the poorest spreadability. The spreadability of GLY and 4% MC bases were found to be similar (*p* < 0.05 relative to the hydrogel with 4% ALG).

### 3.2. Sensory Evaluation

Sensory evaluation parameters of the prepared hydrogels are presented in Table 3, with the scale shown in Table 2.

The prepared substrates, comprising 4% ALG, GLY, 4% MC, and 6% MC, exhibited a uniform and homogeneous structure. The smooth and shiny surface imparted an attractive appearance to the formulations. The 4% MC-based substrate exhibited a lighter texture and superior adhesion compared to the 6% MC and GLY-based substrate, as well as a relatively good cushioning effect. With the exception of the 6% MC-based hydrogel, all the formulations demonstrated excellent spreading ability on the skin surface. Following the application of the formulations to the skin, no adverse skin reactions, such as burning sensation or irritation, were observed.

The results of the sensory testing of all bases (4% ALG, GLY, 4% MC, 6% MC) indicated that the hydrogel with 4% MC was the most favorable substrate for further testing. The material exhibited high uniformity and a lighter consistency-compared to the substrate with GLY and the hydrogel based on 6% MC. Additionally, it demonstrated a relatively brilliant cushion effect. Despite its high stickiness, the base was characterized by ease of spreading. The 4% MC-based hydrogel was also distinguished by significantly higher adhesion compared to the other hydrogels. All bases were observed to exhibit a lack of greasiness and oiliness, with the exception of the starch base, which displayed a notable effect due to its high glycerol content.

The introduction of the active substances STH and ACTH into the 4% MC resulted in slight alterations to the sensory properties. The addition of ACTH, STH, and phosvitin led to a slight reduction in the uniformity of the formulations, a decline in adhesion, and an increase in the “cushion effect.” However, the incorporation of ACTH into the hydrogel and the combination of ACTH with phosvitin resulted in a notable decline in spreadability.

### 3.3. Evaluation of Rheological Properties

A decrease in viscosity was observed for all hydrogels as the shear rate increased. The viscosity values obtained at three shear rates (D = 30 s^−1^, D = 60 s^−1^, and D = 100 s^−1^) for the prepared hydrogel formulations are presented in Table 4.

**Figure 2:**
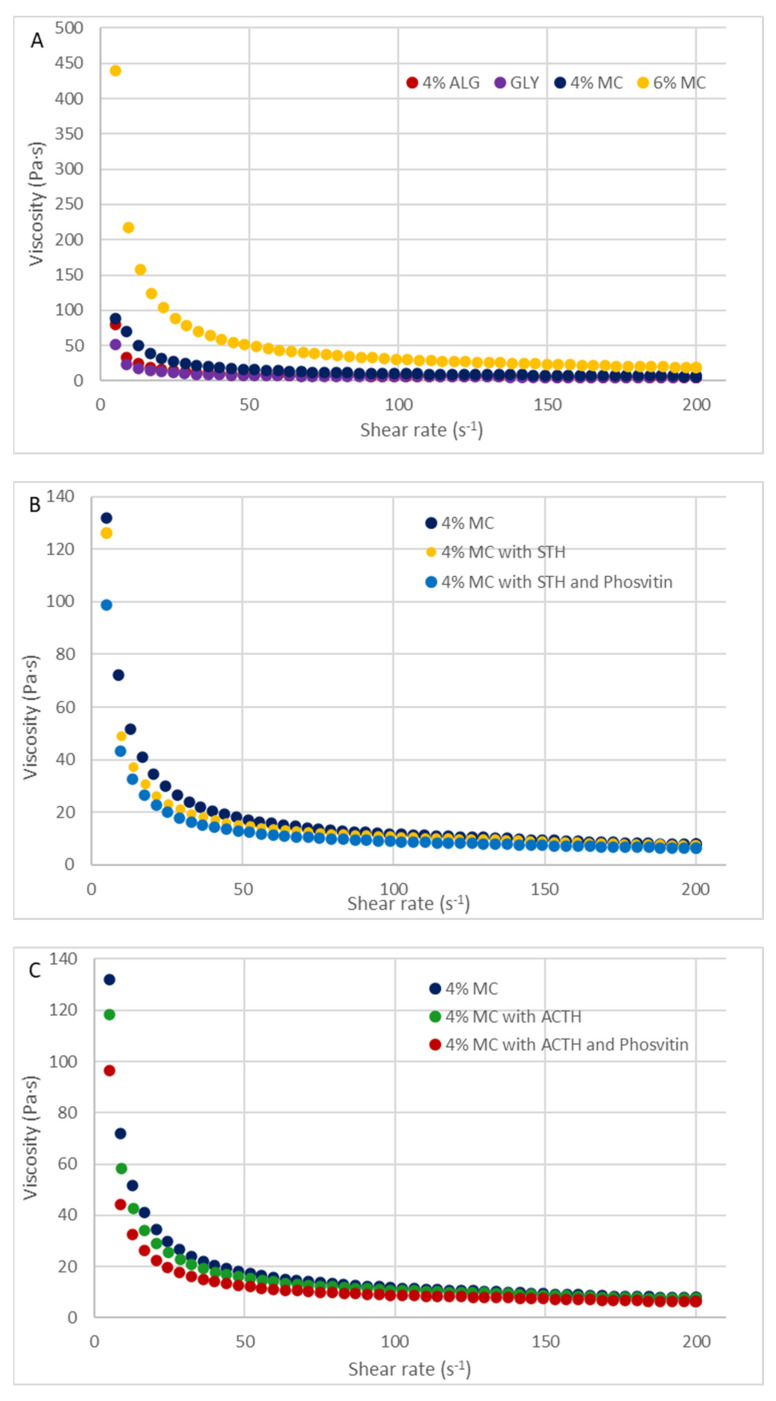
Viscosity curves of base hydrogels: (**A**) 4% ALG, GLY, 4% MC, 6% MC; (**B**) hydrogels with 4% MC, with 4% MC and STH, and with 4% MC, STH, and phosvitin; (**C**) hydrogels with 4% MC, with 4% MC and ACTH and with 4% MC, ACTH and phosvitin.

The hydrogel prepared with 6% MC exhibited the highest viscosity, while the hydrogel prepared with 4% ALG demonstrated the lowest. The viscosity of the hydrogel formulations was found to be as follows, from the lowest to the highest value: for D = 30 s^−1^, ALG < MC 4% < GLY < MC 6%, for D = 60 s^−1^, ALG < GLY < MC 4% < MC 6%, and for D = 100 s^−1^, ALG < GLY < MC 4% < MC 6%. The impact of protein additives on the viscosity of a 4% MC-based hydrogel at three shear rates was assessed. At a shear rate of D = 30 s^−1^, the viscosity of the 4% MC-based hydrogel was 23.78 ± 0.79 Pa·s. The addition of ACTH resulted in an increase in viscosity to 26.25 ± 0.6 Pa·s, while after the addition of phosvitin, the viscosity decreased to a value of 22.72 ± 0.3 Pa·s. For a shear rate of D = 60 s^−1^, the viscosity for the substrate with 4% MC was 16.9 ± 1.48 Pa·s, The addition of ACTH resulted in an increase in viscosity to 17.94 ± 0.41 Pa·s, while the inclusion of phosvitin led to a reduction in viscosity to a value of 16.61 ± 0.47 Pa·s. At a shear rate of D = 100 s⁻^1^, the viscosity value for the hydrogel with 4% MC was 12.26 ± 1.13 Pa·s. ACTH resulted in an increase to 13.51 ± 0.3 Pa·s, which was then followed by a decrease in viscosity value to 12.53 ± 0.8 Pa·s due to the addition of phosvitin. The addition of STH to the hydrogel with 4% MC resulted in an increase in viscosity from 23.78 ± 0.39 to 26.11 ± 0.56 at D = 30 s^−1^. The viscosity increased from 16.9 ± 0.49 to 17.80 ± 0.36 at D = 60 s^−1^ but remained unaltered at D = 100 s^−1^. The addition of phosvitin to hydrogels with STH did not affect the viscosity. The viscosity curves of the prepared formulations are shown in Figure 2.

**Figure 3:**
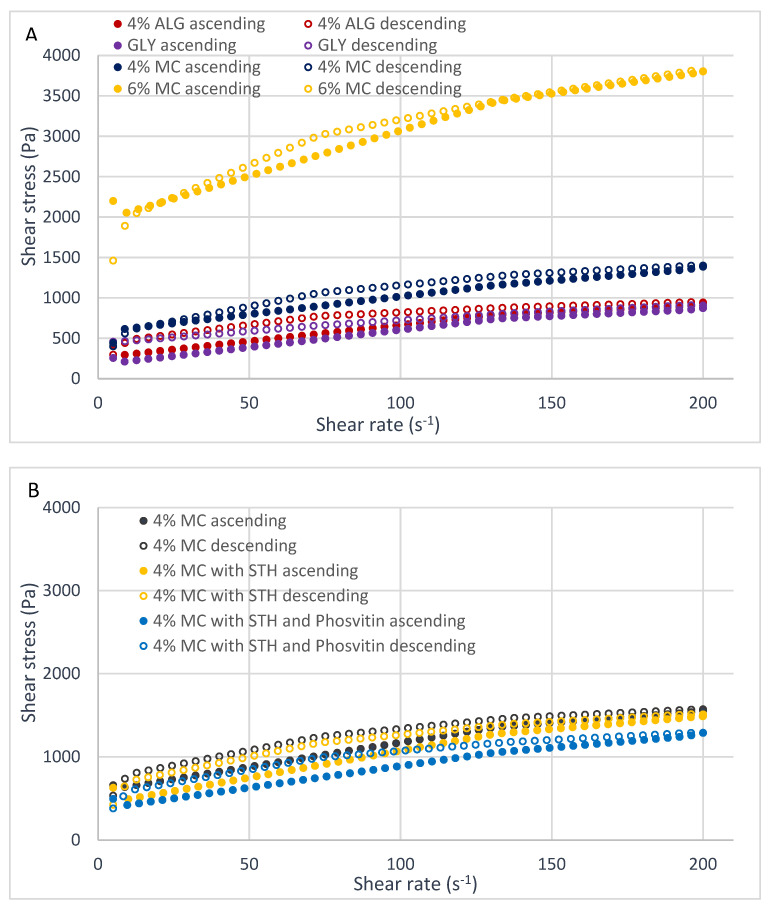

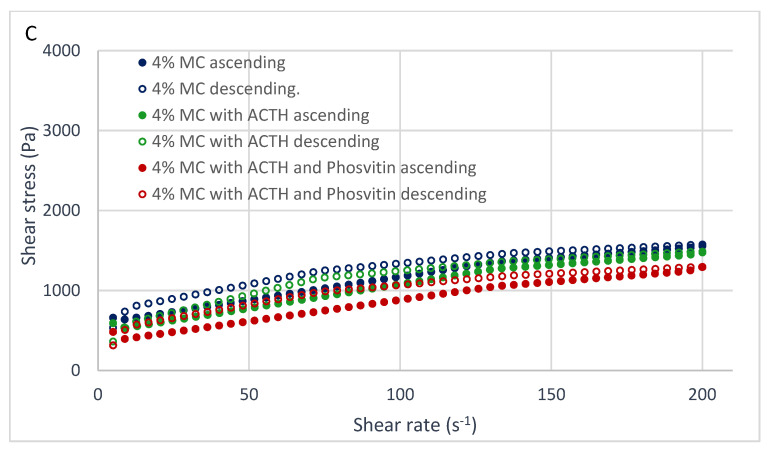
Flow curves obtained by the “FT” method of hydrogels with 4% ALG, GLY, 4% MC and 6% MC (**A**), 4% MC, 4% MC with ACTH and with 4% MC with ACTH and phosvitin (**B**), and 4% MC, 4% MC with STH and 4% MC with STH and phosvitin (**C**), (n = 3).

The prepared hydrogels are classified as non-Newtonian, shear-thinning systems, exhibiting thixotropy. The shape of the viscosity curves indicates the pseudoplastic properties of hydrogels, which are shear-thinning systems (Figure 2). As evidenced by the concavity of the flow curves (Figure 3) their viscosity decreases with increasing shear rate. Viscoplastic systems exhibit a combination of the rheological properties of viscous and elastic bodies, as indicated by the presence of a flow boundary. Additionally, the effect of shear thinning, which causes a bending of the flow curve, is superimposed on this phenomenon.

**Figure 4:**
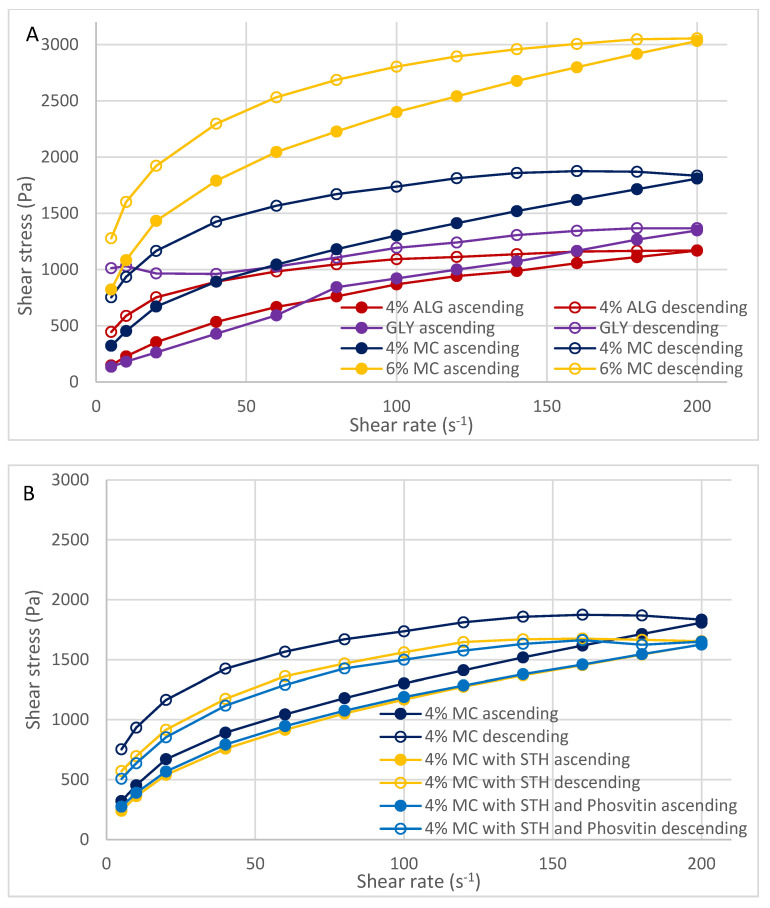

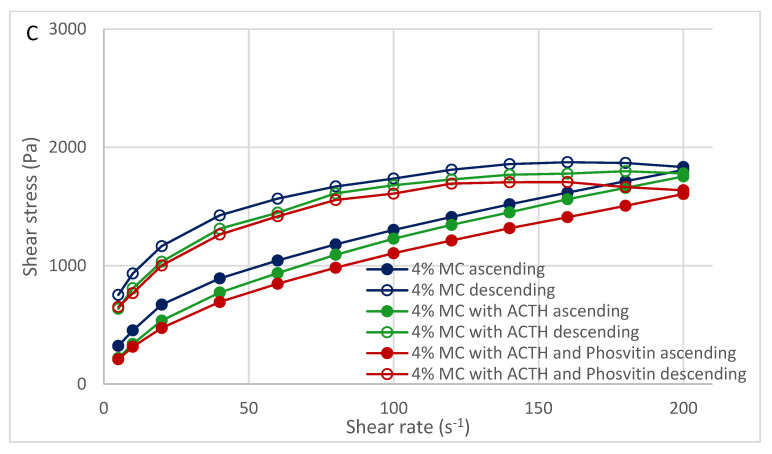
Flow curves (hysteresis loops) from step-by-step test of hydrogels with 4% ALG, GLY, 4% MC and 6% MC (**A**), 4% MC, 4% MC with ACTH and 4% MC with ACTH and phosvitin (**B**), and 4% MC, 4% MC with STH and with 4% MC and STH with phosvitin (**C**) (n = 3).

Figure 3 and Figure 4 illustrate the flow curves obtained through two distinct methodologies: the FT (Flow Test) test (Figure 3) and the SBS (Step-by-Step) test (Figure 4). These figures present the results for hydrogels formulated with three distinct polymers (Figure 3A and Figure 4A), a hydrogel containing 4% MC, a hydrogel containing 4% MC and STH, and a hydrogel the shape of the curves indicates the pseudoplastic properties of the prepared hydrogels, which exhibit shear-thinning behavior. The hydrogels were prepared with 4% MC, with STH and phosvitin (Figure 3B and Figure 4B), and hydrogel with MC 4%, hydrogel with 4% MC and ACTH, and hydrogel with 4% MC with ACTH and phosvitin (Figure 3C and Figure 4C).

In such non-Newtonian systems, where viscosity is dependent upon both shear rate and shear stress, the observed behavior can be attributed to delayed adaptation to flow conditions. The decrease in viscosity at a constant rate until an equilibrium value is established is indicative of the ability to rebuild the original internal structure of prepared hydrogels, which can take from a few seconds to several days.

The hysteresis loop test enables the evaluation of the thixotropic and rheopexy properties of hydrogels. The area of the loop can be used as a measure of thixotropy or rheopexy. The greater the degree of alignment between the ascending and descending curves, the greater the rheological stability of the system. In the case of the hydrogels under study, rheopexy is evident, as the descending curves lie above the ascending curves. The extent of destruction or construction of the internal structure of hydrogels can be determined by determining the areas of the plots between the ascending and descending curves, which form the hysteresis loops. The surface areas of the hysteresis loops of the prepared hydrogels are summarized in Table 5.

It should be noted, however, that the surface area of the hysteresis loop can only be used as a comparative measure of the thixotropic properties of fluids. It is not possible to use this area as an absolute measure of thixotropy, as even for the same hydrogel, the area of the hysteresis loop will be different for different ways of measurement (FT and SBS–“step-by-step”). The addition of protein substances, including ACTH and STH, as well as the addition of phosvitin, resulted in an increase in the area between the ascending and descending curves.

The flow curves from the FT test indicated that the ascending curve was most similar to the descending curve for the hydrogel with 4% MC, and the smallest area of the hysteresis loop was recorded, as confirmed by the calculated value (Table 5). The hydrogel with 4% ALG exhibited the greatest discrepancy between ascending and descending curves, while the hysteresis loop area reached its maximum value. The incorporation of protein-peptide substances exerted a notable influence on the stability of the hydrogel comprising 4% MC, whereas the impact on the hydrogel with 4% ALG was less pronounced. The characteristics of these particles, including their size, may be a contributing factor to the observed loosening of the internal structure of the hydrogels. However, this phenomenon may also facilitate enhanced permeation through these structures.

An analysis of the flow curves obtained in the step test of the dependence of shear stress on shear rate revealed that changes in shear rate do not result in significant alterations in stress due to the addition of STH and ACTH. The addition of ACTH did not significantly affect the rheological stability of the hydrogel with 4% MC. In contrast, the addition of STH and STH with phosvitin appeared to enhance the stability of the hydrogel with 4% MC, as evidenced by a reduction in the hysteresis loop area.

### 3.4. Texture Analysis

The texture study was carried out using a Lamy Rheology TX-700 laboratory texturometer. The obtained texture analysis parameters are presented in Table 6.

The hydrogel containing 6% MC exhibited the highest hardness (0.175 ± 0.008 N), while the hydrogel with starch and glycerol demonstrated a lower hardness (0.150 ± 0.012 N). The hydrogel with 4% MC exhibited the lowest hardness (0.024 ± 0.002 N). The highest value was observed for MC (0.034 ± 0.003 N), while the lowest was recorded for the hydrogel with 4% ALG (0.024 ± 0.002 N). For all hydrogel bases, the difference was statistically significant (*p* < 0.05) when compared to the hydrogel with 4% MC. The incorporation of proteinpeptide compounds, STH and ACTH, into the hydrogel comprising 4% MC exerted a considerable impact on this parameter. The introduction of STH resulted in a 12-fold increase in hardness, while ACTH led to a 13-fold enhancement. Conversely, phosvitin caused a notable reduction in the hardness of the hydrogel with STH, exhibiting a 1.7-fold decrease, and the hydrogel with ACTH, displaying a 1.1-fold reduction.

The hydrogel based on 4% MC exhibited the highest cohesiveness (3.148 ± 0.213), while the gel with GLY demonstrated the lowest (0.835 ± 0.033). The addition of hormonal substances resulted in a notable reduction in the cohesiveness of the hydrogel with 4% MC. The incorporation of STH led to a decrease in cohesiveness by approximately eightfold (0.380 ± 0.090), while ACTH also reduced cohesiveness by a similar extent (0.393 ± 0.083). The addition of phosvitin resulted in a 1.5-fold increase in cohesiveness for preparations with STH (to 0.580 ± 0.027) and a 1.3-fold increase for preparations with ACTH (to 0.525 ± 0.034), with a *p*-value less than 0.05.

The 6% MC-based hydrogel exhibited the highest adhesiveness (1.420 ± 0.084 mJ), while the ALG-based hydrogel demonstrated the lowest (0.200 ± 0.000 mJ). The adhesiveness of the hydrogel with GLY was 0.486 ± 0.038 mJ, while the hydrogel with 4% MC exhibited a lower adhesiveness of 0.357 ± 0.053 mJ.

The incorporation of protein-peptide substances has been observed to enhance the adhesiveness of formulations, thereby prolonging their retention on the skin following application. The addition of STH to the hydrogel increased the adhesiveness with 4% MC to a value of 2.167 ± 0.058 mJ, while the combination of STH with phosvitin resulted in an adhesiveness value of 1.633 ± 0.058 mJ. Similarly, the incorporation of ACTH led to an increase in adhesiveness to 2.2 ± 0.0 mJ, while the combination of ACTH with phosvitin yielded an adhesiveness value of 2.4 ± 0.0 mJ.

**Figure 5:**
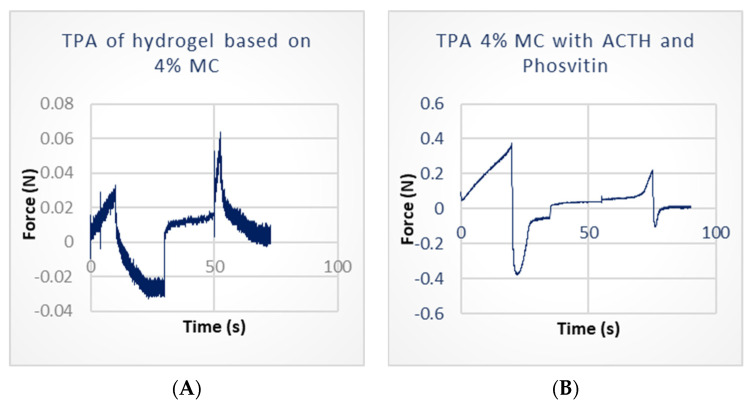
Examples of texture analysis profiles of hydrogel based on 4% MC (**A**) used as carrier and 4% MC with ACTH and phosvitin (**B**).

All hydrogel bases, with the exception of the alginate hydrogel (0.575 ± 0.033), demonstrated comparable elasticity, with values ranging from 1.002 (4% and 6% MC) to 1.094 (GLY). The incorporation of STH and ACTH did not influence the elasticity of the 4% MC hydrogel. Conversely, the addition of phosvitin resulted in a notable reduction in the elasticity of the hydrogel with STH, reaching a value of 0.717 ± 0.016, and a more pronounced decline in the hydrogel with ACTH, with a value of 0.682 ± 0.013 (*p* < 0.05). Selected profiles for the TPA test are presented in Figure 5A,B. Figure 5A displays the texture of the hydrogel with 4% MC, while Figure 5B illustrates the texture of the hydrogel with 4% MC and ACTH and phosvitin.

### 3.5. pH Measurement

The pH values of the obtained hydrogels are presented in Table 7.

The lowest pH value (3.96 ± 0.049) was determined for the 4% MC base, which exhibited a higher pH value (4.51 ± 0.005) than the GLY base, and the highest pH value (6.89 ± 0.042) was observed for the sodium alginate base. Furthermore, the pH of the MC base is contingent upon the concentration of MC. As the concentration of MC increased to 6%, the pH value increased to 4.3 ± 0.021. The addition of STH and ACTH resulted in a notable increase in the pH value of the 4% MC solution, reaching respective values of: The pH value was 5.80 ± 0.021 (STH) and 5.79 ± 0.005. The addition of phosvitin to the hydrogel with STH resulted in a further increase in pH value to 5.94 ± 0.026, whereas in the hydrogel with ACTH, a slight decrease in pH was observed, reaching 5.64 ± 0.0. All prepared preparations with protein-peptide substances exhibited a pH value that was similar to the natural pH of human skin, which is 4.5–6.5. Therefore, these semi-solid forms of the drug can be safely applied to the skin surface without causing irritation.

### 3.6. Study of Permeation through an Artificial Cellulose Membrane and Natural Membrane

**Figure 6:**
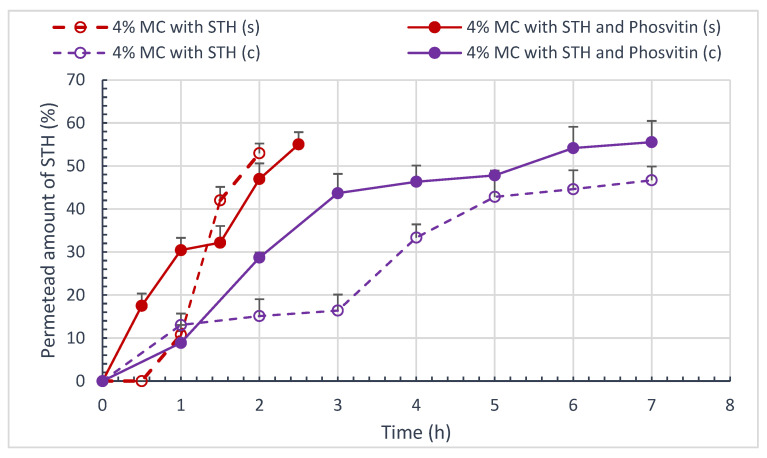
Amount of penetrated STH and STH with phosvitin Q (%) at time t [h] through porcine skin and cellulose membrane; (n = 6).

**Figure 7:**
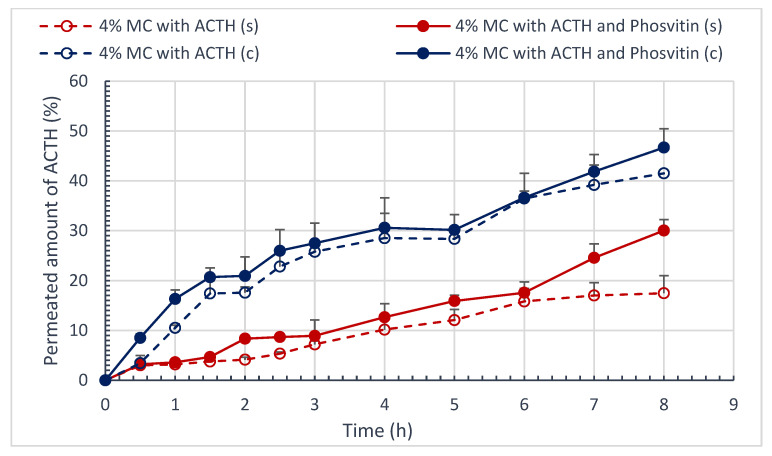
Amount of permeated ACTH and ACTH with phosvitin Q (%) at time T (h) through porcine skin and cellulose membrane; (n = 6).

Figure 6 and Figure 7 present the course of the permeation process of STH and ACTH, as well as the impact of phosvitin on this process through the artificial cellulose membrane and natural porcine skin. Table 8 provides a summary of the percentage of STH and ACTH permeated, along with the calculated AUC areas throughout the permeation process, employing the triangle and trapezoid method (Table 8).

The permeation process exhibits varying dynamics contingent on the membrane utilized. In the case of STH, the permeation process through the artificial cellulose membrane is considerably slower (up to seven hours) than through natural porcine skin (up to 2.5 h). This phenomenon may be attributed to the affinity of STH for natural skin. The addition of phosvitin resulted in a statistically significant increase in the amount of STH permeated through the cellulose membrane, from 46.68 ± 3.21% to 55.56 ± 4.94% (*p* < 0.05). The porcine skin STH permeation study also demonstrated a slight increase in the amount of STH permeated, from 52.96 ± 2.26% to 55.03 ± 2.85%. Phosvitin prolonged the process of STH permeation through porcine skin, extending it from 2 to 2.5 h.

In the ACTH permeation study, a greater quantity of permeated ACTH was observed to traverse the cellulose membrane than to traverse porcine skin. Phosvitin significantly increased the amount of ACTH permeated through porcine skin, from 17.47 ± 3.55% to 30.04 ± 2.18% (*p* < 0.05). To a slightly lesser extent, it increased the amount of ACTH permeated through the artificial membrane from 41.51 ± 3.98% to 46.68 ± 3.78% (*p* < 0.05).

To examine the kinetics of the STH and ACTH permeation process, three kinetic models were employed: the Higuchi model, the Korsmeyer–Peppas model, and the “1” order model. The regression coefficients R^2^, the rate of permeation over time, and the permeation rate constants calculated for “1st” order kinetics for selected hydrogel formulations are presented in Table 9.

The evaluation of the kinetics of the process of STH and ACTH permeation from semi-solid hydrogel formulations does not provide sufficient evidence to select one of the models. The process is dependent on the membrane utilized, exhibiting distinct permeation patterns through artificial and natural membranes, namely the skin. The critical factor is undoubtedly the thickness of the membrane and its structure. The skin is distinguished by its multilayered structure, wherein each layer exhibits a distinct affinity for the hormonal substance applied to its surface. The natural membrane permits the penetration of STH to occur at a considerably faster rate than the artificial membrane, with a fourfold increase in speed. The addition of phosvitin to the cellulose membrane has been observed to enhance the rate of penetration by approximately 1.3 times, whereas, in the case of the skin, the effect is diametrically opposed. The rate of STH permeation through the skin is approximately 1.15 times faster in the absence of phosvitin. The kinetics of STH permeation are most closely aligned with the Higuchi model. ACTH permeates the skin at a slower rate than the cellulose membrane. The rate constant of ACTH permeation through the artificial membrane is approximately 2.8 times higher. The addition of phosvitin has been observed to increase the rate of permeation through both membranes. In the case of the cellulose membrane, the rate is increased by approximately 1.2 times, while in the case of the skin, the rate is increased by approximately 1.9 times. The effect is particularly pronounced in the case of the skin. The kinetics of the ACTH permeation process has been observed to follow a similar pattern according to both the Higuchi and ‘I’ order models.

It is postulated that the release profiles will exhibit similarity when the value of f1 is between 0 and 15, while f2 assumes a value close to 100. The smaller the difference (f1) and the greater the similarity (f2), the more straightforward it is to demonstrate the similarity of the profiles. The similarity and difference coefficients f1 and f2 for formulations containing STH/ACTH and with STH/ACTH with the addition of phosvitin are presented in Table 10. The addition of phosvitin had a significant impact on the permeation profile dissimilarity in all hydrogel systems, with one exception. In the case of ACTH permeation through the cellulose membrane, the f1 coefficient indicated profile similarity, while the f2 coefficient indicated the opposite. While the course of ACTH permeation appears similar to that observed under phosvitin, the amount of ACTH permeated in the presence of phosvitin is 5% greater, representing a statistically significant difference.

### 3.7. Stability Study

The content of the active substances, pH, and viscosity of all prepared formulations remained unaltered for a period of 28 days under the specified storage conditions, which were 25 ± 1 °C and 5 ± 3 °C.

## 4. Discussion

The biodegradable polymers utilized for the preparation of hydrogel substrates as carriers for STH and ACTH were selected based on the authors’ previous studies of the release and permeation of protein-peptide substances and the parameters of the permeation process, as detailed in references [4,38,39,42]. Prior to loading the hydrogel substrates with protein substances, an evaluation of the physicochemical parameters was conducted. This included an assessment of the sensory properties, spreadability, rheological properties, textural properties, and pH values.

A summary of the sensory and physicochemical properties of the obtained hydrogels, based on three different polymers and comprising varying concentrations of sodium alginate (4% ALG), glycerol and wheat starch (GLY), and methylcellulose (4% and 6% MC), was conducted in order to facilitate the selection of an appropriate substrate for further studies. The selected substrate was 4% MC, and the studies were conducted with the inclusion of protein-peptide substances STH and ACTH. In the spreadability test (Figure 1), the 6% MC-based hydrogel had the worst spreadability, and the sodium alginate-based hydrogel had the best; however, in the sensory test (Table 3), it was the 4% MC-based hydrogel that showed the greatest tendency to spread on the skin surface and the greatest stickiness. Of all four substrates, the two substrates based on 4% ALG and 4% MC had more favorable rheological properties. Viscosity, flow curves, and step-by-step flow curves (“step by step”) proved more favorable for 4% ALG and 4% MC; however, the hydrogel with 4% MC had a smaller area between ascending and descending curves in the flow curve “flow test.” This suggests that the hydrogel with 4% MC will more quickly return to its original structure after the removal of shear stress, so it will show greater stability than the hydrogel with 4% ALG. In contrast, the 6% MC-based hydrogel exhibited the highest viscosity at all three shear rates and the highest hardness, which could potentially impede the release of active substances from the prepared hydrogels, leading to an unsatisfactory therapeutic effect. Consequently, this hydrogel was not selected for further investigation.

Analyzing the flow curves obtained by the shear stress-rate dependence step test, changes in shear rate do not induce significant changes in stress due to the addition of STH and ACTH. ACTH did not significantly affect the rheological stability of the hydrogel with 4% MC, while STH and STH with phosvitin even seem to stabilize the hydrogel with 4% MC, as the hysteresis loop areas decreased after the addition of these active substances.

The study used MC with an average viscosity of 3755 mPa·s (2% solution, at 25 °C) at 4% and 6% MC. Tudoroiu et al. studied the properties of MC with different viscosities, among others. They used six commercial varieties of MC, differing in composition and viscosity. Hydrogels were prepared at concentrations of 3% and 4%, and 6%, and 8% with each type of MC: MC 1 with a viscosity of 4000 cPa·s at 2% in water, the second-MC 2, with a viscosity of 1500 cPa·s at 2% in water, and the third MC 3 with a high viscosity, 3000–5000 mPa· s. They found that hydrogels based on cellulose derivatives showed non-Newtonian pseudoplastic behavior, which is a desirable property for semi-solid forms for skin application, contributing to improving their conditioning and spreading on the skin surface, thus promoting topical administration. Considering the experimental data obtained for flow and thixotropic parameters, they concluded that MC 2 hydrogel can be selected at concentrations between 3 and 3.5% for further applications in the field of wound healing [43]. A hydrogel with an MC concentration of 4% and a viscosity of 3755 mPa·s, is intended for use on the skin, so a higher viscosity may provide greater stability.

Gladukh and Podorozhna in their study characterized the rheological profiles of sodium alginate gels as non-Newtonian systems with viscoplastic properties. They found that gels with a sodium alginate concentration of 1.5–2% had the best yield stress, hysteresis loop area, mechanical stability, and flow indices. They pointed out the low degree of destruction of the structural lattice in the process of mechanical action and the presence of thixotropic bonds, in contrast to gels with concentrations of 0.5% and 1%, which were found to be less stable under stress compared to sodium alginate bases with concentrations of 1.5% and 2%. Furthermore, they concluded that the optimal value of mechanical stability was retained by the sodium alginate gel base at a concentration of 2% [44].

On the other hand, Liu et al. in their review summarized that alginate concentrations of 0.5–4% (*w*/*v*) are suitable for cartilage regeneration. Adhesion, colonization, and migration of embedded cells are increased with increasing alginate or Ca^2+^ concentrations, and the effect of alginate and Ca^2+^ concentrations occurs in a dose-dependent manner, while diffusion of large molecules is gradually impeded. Stiffness and compressive modulus increase with increasing alginate concentration, while shear moduli increase with higher Ca^2+^ concentrations. At low Ca^2+^ concentrations, temporary gels can be obtained as high-viscosity solutions, while stable gelation can result from a stable association of crosslinking structures at high Ca^2+^ concentrations [45].

Hydrogels based on biologically inactive alginate, although largely used in wound dressings, are often found in mixtures with other bioactive polymers, such as gelatin, so that better adhesion and cell elongation, desirable in tissue engineering, can be achieved [46,47]. Thus, the validity of choosing 4% MC as a substrate for protein substances was confirmed.

The parameters obtained in the texture analysis indicated better adhesion of 4% MC than 4% ALG and the highest cohesion of 4% MC, which will allow longer contact time with the skin and also greater homogeneity of the 4% MC-based substrate. From the determinations of the pH values of the obtained hydrogels, the value obtained for the substrate with 4% ALG—6.89 ± 0.042 allowed the final choice of the hydrogel with 4% MC (3.86 ± 0.117), since the active protein substances tend to increase the pH value, which was confirmed in an earlier study by Siemiradzka et al. [4], and this could result in causing an irritant effect on the skin when using the substrate with 4% ALG and would require the addition of pH correcting substances.

From the perspective of drug form technology, the substance utilized as a carrier must be cost-effective and readily accessible, fully biocompatible, and ensure optimal drug release. Additionally, it must not impact the drug substance’s shelf life. Proteins, such as albumin, fibrin, phosvitin, and collagen, have been identified as potential biodegradable carriers of active substances.

Song and colleagues investigated the impact of a magnetic field on the synthesis of a phosvitin-microcrystalline cellulose complex in the presence of 1-allyl-3-methylimidazolium chloride. The researchers demonstrated that the prepared hydrogels exhibited a uniform surface structure, adequate swelling capacity, elasticity, and notable thermal stability. The antimicrobial and antioxidant activities of the hydrogels were primarily attributed to the presence of phosvitin and 1-allyl-3-methylimidazolium chloride, respectively [48].

There are few studies in the literature on the permeation of STH and ACTH through model membranes. It is difficult to refer to studies in which the permeation process is further enhanced mechanically, or the skin formulation itself has a modified technology, for example, with the use of nanoparticles. The process of STH permeation from hydrogel forms for skin application was described in the author’s earlier studies [4]. The study looked at the effect of albumin on STH permeation, where it was shown that the best results were obtained with albumin in a ratio of 1:2 to STH. Although higher STH availability was obtained, the base substrate had a different quantitative composition of MC. The hydrogel substrate contained a higher content of MC-6%, while in the current study, MC with a lower concentration of 4% was chosen. In addition, the aim of the study was to evaluate the effect of another protein-phosvitin on the STH permeation process, and the value of the study should be read in the context of comparing the effect of additional substances that can act as permeation promoters to increase the efficiency of the permeation process.

The positive effect of albumin on ACTH permeation was described by Siemiradzka et al. using a glycerol substrate. A study of ACTH permeation through porcine skin showed that albumin can delay or increase and accelerate ACTH permeation. The amount of ACTH released was 58% for the 15 mg/g ACTH hydrogel and 53% for the 20 mg/g concentration. A 1.1-fold increase in ACTH from the lower concentration hydrogel was observed. Adding albumin in a 1:1 ratio to ACTH significantly increased ACTH permeation at the lower concentration (1.4 times). From a hydrogel with a concentration of 20 mg/g ACTH, only after a time of 3 h did albumin increase ACTH penetration, but this change was not statistically significant [47]. In the current study, the amount of ACTH was reduced-to 0.5% (5 mg/g), and the preparation was based on 4% MC. The addition of phosvitin increased the amount of ACTH permeated, and this effect was stronger when natural porcine skin was used-about 2-fold. The differences in the number of protein substances permeated can be attributed to several factors, including the varying particle sizes of these substances, the nature of the substances and membranes, and the affinity of the protein substances for the components of the membranes through which the permeation process is carried out. The permeation process of active substances is also influenced by their particle size. α-phosvitin and β-phosvitin are structures formed by the combination of numerous polypeptides. α-phosvitin contains three or four subunits. Subunits with a molecular weight of 35 to 40 kDa are present in α-phosvitin, while β-phosvitin contains four/five subunits with a weight of 45 kDa [49]. The relatively large number of phosphoryl groups present in the amino acid sequence of phosvitin endows the protein with a hydrophilic character. Due to its high phosphoric acid content resulting from the presence of serine residues, phosvitin can act as a liquid polyanion. Its polyanionic nature (isoelectric point around pH 4) gives phosvitin superior emulsifying activity and enhanced emulsion stability compared to bovine serum albumin, β-casein, and soy protein. The optimal emulsifier is observed at pH 7.0 [7,50]. Both STH and ACTH are protein-peptide substances that exhibit hydrophilic characteristics. At its isoelectric point, STH has a pH of 5.3, and its solubility in water is 1 mg/mL [51]. Similarly, at its isoelectric point, ACTH has a pH range of 4.65 to 4.80, is readily soluble in water (1 mg/mL), and is stable in acidic media [52].

A review of the literature does not permit a definitive conclusion regarding the impact of the drug substance’s hydrophilicity on its release mechanism. The structure of the fibrin matrix is a critical determinant in this process. The primary structure of fibrin offers a substantial opportunity for drug-polymer interactions due to its abundant free amino acid residues. The formation of hydrogen bonds with fibrin has been documented for fluconazole and ciprofloxacin [53,54].

Turek and colleagues posited the potential of utilizing collagen (the primary structural protein of connective tissue) as a vehicle for therapeutic agents. It is distinguished by low antigenicity, biodegradability, biocompatibility, notable hydrophilicity, the capacity for straight forward combination with other polymers, and its adaptability to create diverse forms and formulations. While it has been demonstrated that the hydrophilicity of collagen can result in accelerated solubility and, consequently, an expedited release of the active substance, this is counterbalanced by the intrinsic characteristics of collagen gels, which include augmented viscosity. Collagen has the capacity to stabilize dispersion systems. The introduction of drug substances into the collagen matrix results in the formation of drug-protein bonds, which are primarily ionic, hydrogen, or covalent in nature, depending on the chemical characteristics of the drug substance in question. Furthermore, the structure of collagen enables the “trapping” of the drug substance [55]. A combination of calcium phosphate and type one collagen obtained by 3D printing has demonstrated excellent mechanical properties and can be used in bone tissue engineering [56]. However, the high cost of collagen represents a significant obstacle to its widespread use as a drug carrier.

In this study, Spectra/Por^®^ Dialysis Cellulose Membrane, Biotech CE, MWCO, was employed as a model membrane: 100 kDa, which is distinguished by its exceedingly high degree of purity, the absence of any trace elements, heavy metals, sulfides, or glycerol, its low reactivity, which renders it incapable of adsorbing proteins and liposomes, and its lack of the time-consuming cleaning processes that are typically required. The optimal synthetic membrane for in vitro testing should be chemically inert, should not impede the penetration of drugs, and should facilitate good permeability. Nevertheless, the use of synthetic membranes instead of skin may not reflect the actual penetration of drugs, but rather the release of these substances. In a study conducted by Borge and colleagues on the release of dapsone using a cellulose acetate membrane in a Franz cell, it was observed that the drug release after 24 h of testing was higher than its permeation through the epidermis of the porcine ear [57]. The use of artificial membranes allows for the observation of the product’s ingredients interacting with the skin, which contains keratinocytes and fibroblasts, which are living cells that undergo mitosis and metabolism. However, current skin models, such as EpiDerm™, EpiSkin™, and Labskin™, have demonstrated higher rates of drug molecule penetration compared to human skin [58]. Consequently, when studying the process of STH and ACTH permeation through artificial and natural membranes, there can be notable discrepancies in the quantity of permeated active substances (STH and ACTH) due to the differing membrane behaviors observed.

In addition to synthetic cellulose membranes, there are a variety of scaffolds constructed through computer-aided design (CAD) depending on the desired tissue type. These scaffolds have certain advantages and disadvantages. By selecting appropriate parameters, such as printing speed, atomization speed, and material release rate, it is possible to achieve the desired mechanical and physicochemical properties, as well as a suitable balance and harmony with the intended texture and human body. Bioprinting enables the creation and fusion of multiple multilayered tissues, such as skin.

One of the most prevalent challenges associated with the fabrication of porous scaffolds via 3D bioprinting is the inability to precisely regulate the dimensions and morphology of the pores [56].

The porosity of the matrix is a determining factor in the process of drug substance release, as it increases the surface area of the material. In a porous material, the therapeutic substance is released initially from open pores and subsequently from closed pores. The process is additionally regulated by proteolysis and diffusion. The cross-linking bonds of fibrin matrices also exert a significant influence on the manner in which the drug substance is released. The diameter of fibrins is primarily influenced by the cross-linking time during the formation of endogenous bonds. An increase in fiber diameter has been observed to promote a decrease in polymer matrix cohesion, thereby rendering the system more susceptible to diffusion [59]. The generation of exogenous cross-linking bonds, utilizing glutaraldehyde, tannic acid, or the exposure of polymeric drug carriers, such as fibrin or polypeptides and proteins, can further enhance matrix stability and delay both diffusion and matrix degradation [53,55,60,61,62]. The phenomenon has been documented in fibrin matrices containing tetracycline, daunorubicin, and amphotericin B cross-linked with glutaraldehyde. For instance, crosslinking the fibrin matrix with glutaraldehyde resulted in the biphasic release of the therapeutic substance from the fibrin beads for up to 100 h [53,60].

## 5. Conclusions

The results of the sensory testing, spreadability testing, rheological and textural testing, and pH measurements of all vehicles indicated that the 4% MC-based hydrogel should be selected for further testing. This hydrogel was characterized by high uniformity, a lighter consistency, ease of spreading, and also significantly higher adhesion compared to other hydrogels.

The introduction of the active substances STH and ACTH into the 4% MC hydrogel resulted in slight alterations to the sensory properties. The addition of ACTH, STH, and phosvitin resulted in a slight reduction in the uniformity of the formulations. Furthermore, the addition of ACTH to the hydrogel and ACTH with the addition of phosvitin resulted in a slight reduction in spreadability.

The addition of ACTH did not significantly impact the rheological stability of the hydrogel with 4% MC. Conversely, the incorporation of STH and STH with phosvitin appeared to enhance the hydrogel’s stability.

The introduction of protein-peptide substances causes an increase in adhesiveness, and thus the formulations can last longer on the skin after application.

All prepared formulations with protein-peptide substances exhibited a pH response that was similar to that of human skin, indicating that the addition of pH-modifying substances was unnecessary.

The permeation process exhibits varying dynamics depending on the membrane utilized. In the case of STH, the permeation process through an artificial cellulose membrane is considerably slower (up to 7 h) than through natural porcine skin (up to 2.5 h). The study of STH permeation through porcine skin revealed a slight increase in the amount of STH permeated from 52.96 ± 2.26% to 55.03 ± 2.85. Phosvitin prolonged the process of STH permeation through porcine skin to 2.5 h.

In the study of ACTH permeation through the cellulose membrane, a higher amount of permeated ACTH was observed than through porcine skin. The addition of phosvitin resulted in a significant increase in the amount of ACTH permeated through porcine skin, with a nearly twofold increase observed.

Phosvitin may prove to be a promising permeation promoter for model protein-peptide substances when applied to the skin surface, as oral administration of these substances often results in their loss of activity, while injectable applications frequently cause patients discomfort.

## Figures and Tables

**Table 1:** Summary of components of prepared hydrogel preparations.

Hydrogels Composition (g)	4% ALG	Gly	4% MC	6% MC	4% MC/STH	4% MC/STH/PSV	4% MC/ACTH	4% MC/ACTH/PSV
Wheat starch		8.696						
Methylcellulose			4.0	6.0	4.0	4.0	4.0	4.0
Sodium alginate	4.0							
Glycerol 85%	10.0	13.043	10.0	10.0	10.0	10.0	10.0	10.0
0.5% CaCl_2_ solution	2.0							
STH					0.1	0.1		
ACTH							0.5	0.5
Phosvitin (PSV)						5 × 10^−4^		2.5 × 10^−3^
Water	84.0	78.261	84.0	86.0	85.9	Up to 100.0	85.5	Up to 100.0

**Table 2:** Selected sensory parameters with determination of their degree of intensity.

Scale	5	4	3	2	1
Uniformity	Uniform	Air bubbles	Delicate lumps	Perceptible lumps	Delaminates
Consistency	Very heavy	Heavy	Medium	Light	Very lightweight
Cushion effect	Very large	Large	Medium	Small	Hardly at all
Adhesion	Very good	Good	Medium	Not very good	Lack of adhesion
Spreading	Very difficult to spread	Difficult to spread	Medium spreads	Spreads easily	Very Spreads easily
Stickiness	Very large	Large	Medium	Small	Almost not at all
Greasiness	Leaves greasy film	Quite greasy	Medium Oily	Slightly greasy	No film

**Table 3:** The parameters of sensory evaluation (score on a scale of 1–5).

Parameter	4% ALG	GLY	4% MC	6% MC	4% MC/STH	4% MC/STH/PSV	4% MC/ACTH	4% MC/ACTH/PSV
Uniformity	5	5	5	5	4	4	4	4
Consistency	3	4	3	5	3	3	3	3
Cushion effect	3	1	3	5	4	4	4	4
Adhesion	3	2	5	1	3	3	3	3
Spreading	4	4	4	2	4	4	3	3
Stickiness	2	1	4	5	4	4	4	4
Greasiness	1	2	1	1	1	1	1	1

A linear structured scale with the following intensity determinations: 1—none, 2—very weak, 3—weak, 4—medium, 5—strong.

**Table 4:** The viscosity (ƞ) of the prepared base hydrogels and hydrogels with 4% MC and STH, 4% MC with STH and phosvitin, 4% MC and ACTH, and 4% MC with ACTH and phosvitin at three constant shear rates: D = 30 s^−1^, D = 60 s^−1^ and D = 100 s^−1^ (n = 6).

Formulation Code	Viscosity η (Pa·s)		
	D = 30 s^−1^	D = 60 s^−1^	D = 100 s^−1^
4% ALG	17.23 ± 1.10 ^1,4^	11.37 ± 0.38 ^1,2,4^	8.01 ± 0.40 ^1,2,3,4^
GLY	28.83 ± 1.85 ^1,4^	12.56 ± 2.54 ^1,2,4^	8.63 ± 0.51 ^1,2,4^
4% MC	23.78 ± 0.39	16.90 ± 0.49 ^2^	12.26 ± 1.13 ^2,3^
6% MC	66.06 ± 2.89 ^1,4^	39.77 ± 0.58 ^1,2,4^	26.73 ± 1.70 ^1,2,3,4^
4% MC/STH	26.11 ± 0.56 ^1,4^	17.80 ± 0.36 ^2^	12.27 ± 0.29 ^2,3^
4% MC/STH/PSV	23.83 ± 0.45	17.06 ± 1.35 ^2^	12.18 ± 0.23 ^2,3^
4% MC/ACTH	26.25 ± 0.60 ^1,4^	17.94 ± 0.41 ^2^	13.51 ± 0.30 ^2,3,4^
4% MC/ACTH/PSV	22.72 ± 0.30 ^1^	16.61 ± 0.47 ^2^	12.53 ± 0.80 ^2,3^

^1^—statistically significant difference with respect to the substrate with 4% MC at shear rates of D = 30 s^−1^, D = 60 s^−1^, and D = 100 s^−1^ (*p* < 0.05). ^2^—statistically significant difference with respect to shear rate of 30 s^−1^. ^3^—statistically significant difference with respect to shear rate of 60 s^−1^. ^4^—statistically significant difference with respect to the substrate with 4% MC at shear rates of D = 30 s^−1^, D = 60 s^−1^, and D = 100 s^−1^ (*p* < 0.05).

**Table 5:** AUC of the flow curves obtained by FT and SBS test of made hydrogels preparations.

Formulation Code	$\mathrm{AUC}\;\mathrm{of}\;\mathrm{the}\;\mathrm{Hysteresis}\;\mathrm{Loop}\;(\frac{N}{m^{2}}\cdot s^{-1})$	
	FT Test	SBS Test
4% ALG	7321.92	226.44
GLY	1185.56	468.46
4% MC	514.41	352.77
6% MC	732.50	347.33
4% MC/STH	1214.21	279.84
4% MC/STH/PSV	1483.00	213.13
4% MC/ACTH	729.38	345.95
4% MC/ACTH/PSV	874.21	373.89

**Table 6:** Texture analysis parameters of prepared hydrogel formulations; (n = 6).

Formulation Code	Hardness [N]	Cohesiveness	Adhesiveness [mJ]	Elasticity
4% ALG	0.024 ± 0.002 ^1^	1.764 ± 0.191 ^2^	0.200 ± 0.000 ^3^	0.575 ± 0.033 ^4^
GLY	0.150 ± 0.012 ^1^	0.835 ± 0.033 ^2^	0.486 ± 0.038 ^3^	1.094 ± 0.077
4% MC	0.034 ± 0.003	3.148 ± 0.213	0.357 ± 0.053	1.002 ± 0.001
6% MC	0.175 ± 0.008 ^1^	2.824 ± 0.046	1.420 ± 0.084 ^3^	1.002 ± 0.001
4% MC/STH	0.401 ± 0.029 ^1^	0.380 ± 0.090 ^2^	2.167 ± 0.058 ^3^	1.001 ± 0.001
4% MC/STH/PSV	0.237 ± 0.010 ^1^	0.580 ± 0.027 ^2^	1.633 ± 0.058 ^3^	0.717 ± 0.016 ^4^
4% MC/ACTH	0.439 ± 0.052 ^1^	0.393 ± 0.083 ^2^	2.200 ± 0.000 ^3^	1.001 ± 0.000
4% MC/ACTH/PSV	0.387 ± 0.016 ^1^	0.525 ± 0.034 ^2^	2.400 ± 0.000 ^3^	0.682 ± 0.013 ^4^

^1^—statistically significant difference with respect to the hardness of the hydrogel with 4% MC (*p* < 0.05). ^2^—statistically significant difference with respect to the cohesiveness of the 4% MC hydrogel (*p* < 0.05). ^3^—statistically significant difference with respect to the adhesiveness of the 4% MC hydrogel (*p* < 0.05). ^4^—statistically significant difference with respect to the elasticity of the 4% MC hydrogel (*p* < 0.05).

**Table 7:** Average the pH values with SD (n = 3).

Formulation Code	1	2	3	Average ± SD
4% ALG	6.85	6.89	6.93	6.89 ± 0.042 ^1^
GLY	4.51	4.51	4.52	4.51 ± 0.005 ^1^
4% MC	4.02	3.94	3.93	3.96 ± 0.049
6% MC	4.33	4.28	4.30	4.30 ± 0.021 ^1^
4% MC/STH	5.82	5.81	5.78	5.80 ± 0.021 ^1^
4% MC/STH/PSV	5.97	5.93	5.92	5.94 ± 0.026 ^1^
4% MC/ACTH	5.80	5.79	5.79	5.79 ± 0.005 ^1^
4% MC/ACTH/PSV	5.64	5.64	5.64	5.64 ± 0.000 ^1^

^1^—statistically significant difference with respect to hydrogel based on 4% MC.

**Table 8:** The mean amounts of permeated STH and STH with phosvitin and ACTH and ACTH with phosvitin Q (%) at time (h) through porcine skin and cellulose membrane; (n = 6).

Time [h]	The Total Amount of STH/ACTH Penetrated (%)			
	STH	STH with PSV	STH	STH with PSV
	**Porcine Skin**		**Cellulose Membrane**	
2	52.96 ± 2.26			
2.5	-	55.03 ± 2.85 ^1^		
7	-	-	46.68 ± 3.21	55.56 ± 4.94 ^1^
AUC_(0-nh)_	53.337 ± 4.12	122.267 ± 11.81 ^2^	97.565 ± 6.28	107.744 ± 8.57 ^2^
	**ACTH**	**ACTH with PSV**	**ACTH**	**ACTH with PSV**
8	17.47 ± 3.55	30.04 ± 2.18 ^3^	41.51 ± 3.98	46.68 ± 3.78 ^3^
AUC_(0–8h)_	78.94 ± 11.73	106.65 ± 12.80 ^4^	208.49 ± 18.04	229.43 ± 22.35 ^4^

^1^—Statistically significant difference with respect to the amount of STH permeated (*p* < 0.05). ^2^—Statistically significant difference with respect to AUC of STH (*p* < 0.05). ^3^—Statistically significant difference with respect to the amount of ACTH permeated (*p* < 0.05). ^4^—Statistically significant difference with respect to AUC of ACTH (*p* < 0.05).

**Table 9:** STH and ACTH permeation R^2^ regression coefficients, permeation rates, and rate permeation constants for first order kinetic for the made formulations.

Active Substance	R^2^			Permeation Rate [mg/cm^2^/h]	First Order Rate Permeation
	Higuchi’s Model	Korsmeyer–Peppas Model	First Order Model		
Porcine skin					
STH	0.9406	0.9039	0.9480	0.112 ± 0.005	-
STH/PSV	0.9493	0.9622	0.8813	0.097 ± 0.005 ^1^	-
ACTH	0.9358	0.9692	0.9786	0.048 ± 0.007	0.024 ± 0.004
ACTH/PSV	0.9059	0.9467	0.9576	0.068 ± 0.006 ^2^	0.0447 ± 0.006 ^2^
Cellulose membrane					
STH	0.9571	0.9443	0.9588	0.029 ± 0.005	-
STH/PSV	0.9549	0.9171	0.8866	0.038 ± 0.006 ^1^	-
ACTH	0.9735	0.9177	0.9526	0.081 ± 0.004	0.0670 ± 0.003
ACTH/PSV	0.9654	0.961	0.9786	0.099 ± 0.002 ^2^	0.0786 ± 0.006 ^2^

^1^—Statistically significant difference with respect to the rate of STH penetration (*p* < 0.05). ^2^—Statistically significant difference with respect to the rate of ACTH penetration (*p* < 0.05).

**Table 10:** Pairwise comparison of the similarity factors f1 and f2 of the STH and ACTH release profiles.

Preparation	f1	f2	Dissolution Profile
Cellulose membrane permeation profiles			
STH/PSV vs. STH	28.56	174.43	Dissimilar
ACTH/PSV vs. ACTH	12.63	128.15	Similar/Dissimilar
Porcine skin permeation profiles			
STH/PSV vs. STH	102.15	176.68	Dissimilar
ACTH/PSV vs. ACTH	39.26	135.25	Dissimilar

STH—MC (4%) with STH (0.1%). STH/PSV—MC (4%) with STH (0.1%) and Phosvitin (0.05%). ACTH—MC (4%) with ACTH (0.5%). ACTH/PSV—MC (4%) with ACTH (0.5%) and Phosvitin (0.25%).

## Data Availability

The original contributions presented in the study are included in the article, further inquiries can be directed to the corresponding author.
